# New online-only Case Reports - May listing

**Published:** 2012-05

You can access the case report and trainee presentations by using your College-issued Athens username and password to enter the members’ area of the College website (www.rcseng.ac.uk/members/resources/annals) and the following link to the Annals.

**Adenoid cystic carcinoma of the breast: case report and review of literature**

R Veeratterapillay, S Veeratterapillay, E Ward, H Rhout, T Fasih

doi 10.1308/003588412X13171221499829

**Local anaesthetic surgical treatment of severe objective pulsatile tinnitus: a useful technique**

AA Aikoye, TY Tang, FJ Meyer

doi: 10.1308/003588412X13171221498820

**Primary extraskeletal Ewing sarcoma involving the carotid artery: a case report and review of the current literature**

R Rallala, D Nikkhah, P Nix, C Woodhead

doi 10.1308/003588412X13171221501348

**A perforated duodenal ulcer presenting as inferior lead ST elevation following amphetamine use**

HG Jones, L Hopkins, A Clayton, E McRain

doi: 10.1308/003588412X13171221588659

**Primary angiosarcoma of the larynx: a rare entity**

R Ratna, A Deshmukh, E Sridhar, D Chaukar, A D’Cruz

doi: 10.1308/003588412X13171221588776

**The oldest reported mirror hand? The curse of the coal-house frog!**

NJ Marsden, IS Whitaker, DE Royce

doi: 10.1308/003588412X13171221589577

**Urinary tract transitional cell carcinoma and melanosis of the bladder: a case report and review of the literature**

JA Harikrishnan, S Chilka, S Jain

doi: 10.1308/003588412X13171221589135

**Bilateral lower limb compartment syndrome: a potentially destructive complication of breast reduction**

M Malahias, S Ghorbanian, P Lemonas

doi: 10.1308/003588412X13171221589379

**Transnasal K-wire to stabilise a complex zygomatic fracture: a forgotten method of fixation**

MR Singh, R Shekar, T Flood, A Greenstein

doi: 10.1308/003588412X13171221590494

**A case report of anaphylaxis to chlorhexidine during urinary catheterisation**

J Noel, A Temple, GJA Laycock

doi: 10.1308/003588412X13171221590610

**Curtain gastroplasty as an option in high risk emergency hiatus hernia repair**

JS Pollard, CJ Lewis

doi: 10.1308/003588412X13171221590773

**Spontaneous liver haemorrhage and haemobilia as initial presentation of undiagnosed polyarteritis nodosa**

N Rattula, D Tsapralis, M Morgan, D Mirza

doi: 10.1308/003588412X13171221590737

**SIADH following laparoscopic (totally extraperitoneal) inguinal hernia repair**

M Ahmed, J Pattar

doi: 10.1308/003588412X13171221591051

**A rare case of perforated caecum after acute pancreatitis**

LCE Martin, M Stavrou, F El-Madani, V Naik, R Jain, S Gupta

doi: 10.1308/003588412X13171221590692

**Small bowel obstruction induced by spontaneous partial deflation of an intragastric balloon**

VS Hegade, R Sood, AC Douds

doi: 10.1308/003588412X13171221590539

**Prolonged survival after surgical management of metastases in malignant melanoma: three case reports**

SR Whitaker, SJ Houston

doi: 10.1308/003588412X13171221590458

**Subacute reaction to endoscopic mucosal resection mimicking perforation**

JML Williamson, P Dunkley, D Hewin

doi: 10.1308/003588412X13171221590412

